# Personalised genomic strategies improve diagnostic yield in inherited retinal dystrophies: a stepwise, patient-centred approach

**DOI:** 10.1038/s41433-025-03981-1

**Published:** 2025-09-09

**Authors:** Anna Esteve-Garcia, Ariadna Padró-Miquel, Jaume Català-Mora, Cristina Sau, Delia Yubero, Zelia Corradi, Frans P. M. Cremers, Pilar Barberán-Martínez, José M. Millán, Gema García-García, Ilyana Ismael, Luis Arias, Estefania Cobos, Cinthia Aguilera

**Affiliations:** 1 Clinical Genetics Unit, Metropolitan South Clinical Laboratory, Bellvitge University Hospital, Institut d’Investigació Biomèdica de Bellvitge (IDIBELL), L’Hospitalet de Llobregat, Barcelona, Spain; 2 PhD Program in Genetics, Faculty of Biology, University of Barcelona, Barcelona, Spain; 3 Genetics Laboratory, Metropolitan South Clinical Laboratory, Bellvitge University Hospital, Institut d’Investigació Biomèdica de Bellvitge (IDIBELL), L’Hospitalet de Llobregat, Barcelona, Spain; 4 Department of Ophthalmology, SJD Barcelona Children’s Hospital, Esplugues de Llobregat, Barcelona, Spain; 5 Hereditary Retinal Dystrophies Unit, Ophthalmology Departments, SJD Barcelona Children’s Hospital and University Hospital of Bellvitge, Barcelona, 08950 Spain; 6 Centre for Biomedical Research on Rare Diseases (CIBERER), Instituto de Salud Carlos III, Madrid, Spain; 7 Genetics Department, Hospital Sant Joan de Déu and Institut de Recerca Sant Joan de Déu, Barcelona, Spain; 8 Department of Human Genetics, Radboud University Medical Center, Nijmegen, the Netherlands; 9 Molecular, Cellular, and Genomic Biomedicine Group, IIS-La Fe, Valencia, Spain; 10 University and Polytechnic La Fe Hospital of Valencia, Valencia, Spain; 11 Department of Ophthalmology, Bellvitge University Hospital, Institut d’Investigació Biomèdica de Bellvitge (IDIBELL), L’Hospitalet de Llobregat, Barcelona, Spain

**Keywords:** Disease genetics, Hereditary eye disease

## Abstract

**Background:**

Inherited retinal dystrophies (IRDs) are a genetically heterogeneous group of conditions, with approximately 40% of cases remaining unresolved after initial genetic testing. This study aimed to assess the impact of a personalised genomic approach integrating whole-exome sequencing (WES) reanalysis, whole-genome sequencing (WGS), customised gene panels and functional assays to improve diagnostic yield in unresolved cases.

**Subjects/Methods:**

We retrospectively reviewed a cohort of 597 individuals with IRDs, including 525 probands and 72 affected relatives. Among the 221 genetically unresolved cases, a subset of 101 was selected for stepwise re-evaluation. This included WES reanalysis with updated virtual panels, WGS in selected cases and targeted sequencing of complex regions. Variant interpretation was refined using updated classification criteria, segregation analysis and functional assays such as mRNA and minigene/midigene studies.

**Results:**

An initial diagnostic yield of 59.6% (313/525) was achieved through first-tier genetic testing. Re-evaluation of the 101 prioritised cases resulted in 42 new diagnoses in probands and resolution of 7 more familial cases, yielding 49 additional diagnoses among previously unresolved patients (48.5%). This increased the overall diagnostic rate for probands to 67.6% (355/525). Functional assays confirmed pathogenicity of variants in *ABCA4*, *ATF6*, *REEP6,* and *TULP1*, while WGS enabled the detection of structural and deep intronic variants, further enhancing diagnostic accuracy.

**Conclusions:**

A patient-centred, stepwise genomic approach significantly improved the molecular diagnosis of IRDs. This strategy supports the clinical utility of periodic WES reanalysis and targeted use of customised panels, WGS and functional assays. The proposed workflow is scalable and applicable to routine clinical practice, contributing to precision medicine in IRDs.

## Introduction

Inherited retinal dystrophies (IRDs) are a leading cause of blindness worldwide, characterised by extensive genetic heterogeneity that complicates molecular diagnosis [1–3]. To date, pathogenic variants in over 300 genes have been implicated in IRDs (RetNet, https://web.sph.uth.edu/RetNet/; accessed on 3 February 2025), affecting both coding and non-coding regions. These include deep intronic variants (e.g., *ABCA4*, *CEP290* and *USH2A*), GC-rich regions (*RPGR*-ORF15) and structural variants (SVs) such as large deletions and complex rearrangements [4–10].

Next-generation sequencing (NGS), particularly whole-exome sequencing (WES) and gene panels, has transformed IRD diagnostics by enabling simultaneous analysis of multiple genes [11–13]. However, despite these advancements, a significant proportion of cases remain unresolved, with diagnostic yields ranging from 49% to 75%, leaving nearly 40% of patients without a molecular diagnosis [3, 12–14]. This highlights key limitations of current NGS approaches: gene panels fail to capture non-targeted regions and WES has limited sensitivity for deep intronic variants [15]. Additionally, both methods struggle with SVs detection and pathogenic variants in repetitive or homologous regions [15]. Whole-genome sequencing (WGS) provides a more comprehensive analysis, covering both coding and non-coding regions and allowing the identification of complex genomic rearrangements often missed by WES [16–20].

Beyond sequencing, variant interpretation remains a challenge, with many variants classified as variants of uncertain significance (VUS), complicating clinical decision-making [2, 21]. Emerging research continues to refine variant classification, revealing that synonymous and hypomorphic variants, previously considered benign, can contribute to IRD pathogenesis [22, 23]. This highlights the need for periodic WES reanalysis and refinements to American College of Medical Genetics and Genomics and the Association for Molecular Pathology (ACMG-AMP) guidelines to improve diagnostic accuracy [24–26].

Functional validation is critical for confirming variant pathogenicity, particularly in non-coding regions. However, the inaccessibility of retinal tissue remains a key challenge [27]. In vitro assays, such as mRNA analysis and minigene/midigene assays, have emerged as powerful tools to elucidate IRD mechanisms and improve variant interpretation [28, 29].

This study focuses on a subset of 101 unresolved cases, selected from a larger diagnostic cohort, to assess the impact of a personalised, case-by-case re-evaluation strategy. By integrating WES reanalysis, customised gene panels, WGS, functional assays and updated classification frameworks, this approach aims to enhance the diagnostic yield for IRDs. Beyond improving molecular diagnosis, this strategy contributes to a better understanding of IRD pathogenesis, facilitates access to gene-targeted therapies and enables more accurate genetic counselling.

## Subjects and methods

### Cohort description

We retrospectively reviewed the clinical and genetic records of 597 adult patients with a confirmed clinical diagnosis of IRD, all monitored at the Hereditary Retinal Dystrophies Unit of Bellvitge University Hospital. This cohort included 525 probands from unrelated families and 72 affected relatives (familial cases). Clinical diagnoses were based on comprehensive ophthalmological assessments, including fundus examination, optical coherence tomography, autofluorescence imaging and electrophysiology when indicated. All individuals underwent genetic testing between 2021 and 2024, primarily through targeted gene panels or WES, both of which included copy number variant (CNV) analysis as part of standard diagnostic protocols.

Among the 525 probands, 221 remained without a conclusive molecular diagnosis after initial testing. Based on clinical presentation, family history and previous genetic findings, a subset of 101 unresolved cases were selected for further re-evaluation. This subgroup constitutes the primary study population in which the personalised genomic approach described in this study was applied.

### Genetic testing workflow

Initially, cases were classified as resolved or unresolved based on prior genetic results. From the 221 unresolved cases, a subset of 101 cases was selected for stepwise, case-by-case genetic re-evaluation. This re-assessment involved one or more of the following approaches: variant reinterpretation and reclassification, WES reanalysis with updated virtual panels, WGS, customised gene panels or functional studies, depending on the specific characteristics of each case. This additional testing was performed between 6 months and 3 years after the initial analysis, depending on the clinical course, newly available evidence and the implementation of updated sequencing tools.

Case prioritisation was based on both clinical and genetic criteria, including reproductive planning, family history of IRDs, the presence of a single pathogenic variant in recessive genes with a consistent phenotype, and cases with no candidate variants in which the initial study had been performed over a year earlier—especially when the original panel might not have included recently associated IRD genes. Notably, timing alone was not the sole determinant; in several cases, prioritisation was driven more by the nature of preliminary findings than by the time elapsed since the first analysis. Exclusion criteria included patient mortality, lack of clinical follow-up or reclassification of the phenotype as non-IRD.

## WES reanalysis

Unresolved cases underwent WES reanalysis using an updated IRD gene panel for non-syndromic cases (Supplementary Data) and a phenotype-driven approach with Human Phenotype Ontology (HPO)-guided analysis for syndromic IRDs [30]. Updated annotation tools were applied in both analyses, with bioinformatics performed using Datagenomics software (versions 19.1 and 22.4.0) and CNV detection was carried out via the VarSeq platform (Golden Helix).

## Whole-genome sequencing analysis and customised gene panel sequencing

WGS was performed using the KAPA HyperPrep Kit (Roche) and the xGen DNA Library Prep EZ Kit (Integrated DNA Technologies), with sequencing conducted on the Illumina NovaSeq 6000 platform. Bioinformatics analysis was carried out using the CNAG (Centro Nacional de Análisis Genómico) GPAP (Genome-Phenome Analysis Platform, hg19) and Emedgene (Illumina, hg19) platforms. Variants were filtered using an expanded IRD panel that also included candidate IRD genes (Supplementary Data) [17, 31]. A customised gene panel targeting *ABCA4* deep intronic regions and *RPGR*-ORF15 repetitive region was processed using the Agilent SureSelect XT HS2 and the Magnis NGS Prep system (Agilent Technologies, CA, USA), sequenced on the Illumina MiSeq platform and analysed using the Datagenomics software.

## Variant filtering and classification

Variants with read depth >20x and an allele frequency ≥20% were considered, except for *RPGR*-ORF15, where all variants were retained. A minor allele frequency threshold of 0.05 in gnomAD v2.1.1 [32] was applied, prioritising deleterious variants, including nonsense, frameshift, splice site and missense variants. Pathogenicity was assessed using REVEL [33] for missense variants and SpliceAI [34] for splicing impact. Variants were classified according to the ACMG-AMP classifications standards [24], the latest recommendations from the Sequence Variant Interpretation Working Group (SVI-WG) [35] and gene-specific adaptations, such as those from Cornelis et al. (2023) for *ABCA4* gene [36].

## Validation of variants and splice site assays

Variants were validated using Sanger sequencing, digital PCR, array-CGH or MLPA, depending on variant type, following standard protocols. Splicing impact was assessed through mRNA analysis and minigene/midigene assays.

To evaluate the impact of *REEP6* c.349-4G>T and c.349-1G>A variants, as well as the *ATF6* c.160-8A>G variant, RNA was extracted from nasal ciliary cells (*REEP6*) and whole blood (*ATF6*) using the RNeasy Mini Kit (Qiagen) and Maxwell® RSC SimplyRNA Blood Kit (Promega), respectively. cDNA synthesis was performed using the PrimeScript RT Reagent Kit (TaKaRa), followed by PCR amplification with primers listed in Supplementary Table S1. PCR products were purified (ExoSAP-IT, Applied Biosystems) and analysed by Sanger sequencing (BigDye Terminator v3.1, Applied Biosystems). Electropherograms were analysed using Mutation Surveyor v5.1.2 (for *ATF6*) and FinchTV (for *REEP6*) software. The potential protein impact of these variants was assessed using Expasy translate tool [37].

The splicing effect of *ABCA4* c.859-442C>T variant was investigated using an in vitro splice assay based on a previously established wild-type midigene (BA7) containing *ABCA4* exons 7 to 11 [38]. The variant was introduced via site-directed mutagenesis using oligonucleotides listed in Supplementary Table S2. Wild-type and mutant constructs were transfected into HEK293T cells, followed by RNA extraction (Nucleospin RNA, Machery-Nagel) and cDNA synthesis (iScript, Bio-Rad). RT-PCR was performed using *ACTB* and *RHO* exon 5 as controls. Splicing defects were analysed via electrophoresis, Sanger sequencing and semi-quantitative mRNA analysis using Fiji software. Further details on the midigene assay are provided in the Supplementary Material.

Additionally, a minigene splice assay for *TULP1* c.822G>T was conducted as previously described [39].

### Ethical considerations

This study was approved by the Research Ethics Committee of Bellvitge University Hospital (reference number PR014/22) and conducted in accordance with the Declaration of Helsinki [40]. Informed consent was obtained from all participants and biological samples were sourced from the Biobank HUB-ICO-IDIBELL, part of the ISCIII Biobanks and Biomodels Platform when needed.

## Results

### Cohort characterisation

**Fig. 1:**
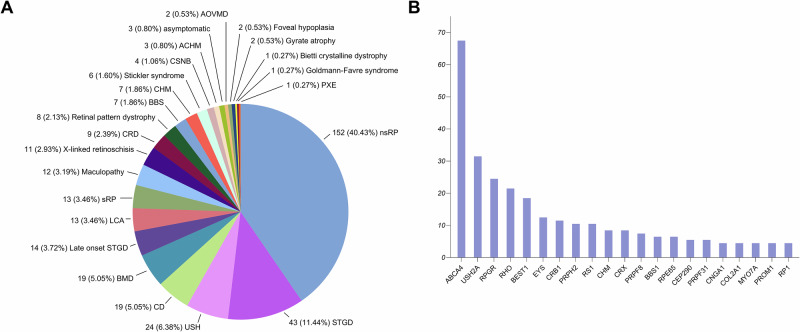
Clinical and genetic distribution in resolved cases. **A** The most common clinical diagnoses in the genetically resolved cohort (*n* = 376) were non-syndromic retinitis pigmentosa (nsRP, 40.4%, 152/376), Stargardt disease (STGD, 11.4%, 43/376) and Usher syndrome (USH, 6.3%, 24/376). **B** Pathogenic variants were identified in 70 genes across 24 IRD subtypes. Only genes implicated in ≥5 cases are shown; the remaining 49 genes not shown were found in fewer than 5 cases. The most frequently affected genes were *ABCA4* (18.1%, 68/376), *USH2A* (8.5%, 32/376), and *RPGR* (6.7%, 25/376). CD cone dystrophy, BMD best macular dystrophy, LCA leber congenital amaurosis, sRP syndromic retinitis pigmentosa, CRD cone-rod dystrophy, BBS Bardet-Biedl syndrome, CHM choroideremia, CSNB congenital stationary night blindness, ACHM achromatopsia, AOVMD adult-onset vitelliform macular dystrophy, PXE pseudoxanthoma elasticum.

Of the 597 cases, 376 were classified as genetically resolved (P1-P376 in Supplementary Table S3), including 313 probands and 63 familial cases. This cohort exhibited a near-equal sex distribution (189 females and 187 males). The mean age of symptom onset was 23.3 years (range: 1 to 75 years), with 51.1% (192/376) of patients reporting a family history of IRD. Pathogenic variants were identified in 70 genes across 24 IRD subtypes (Fig. 1). Among the 525 probands tested, first-tier genetic testing achieved a diagnostic yield of 59.6% (313/525).

From the 221 unresolved cases, a subset of 101 was selected for personalised reanalysis based on clinical and genetic prioritisation criteria. This group constitutes the primary study population.

### Diagnostic improvement

**Fig. 2:**
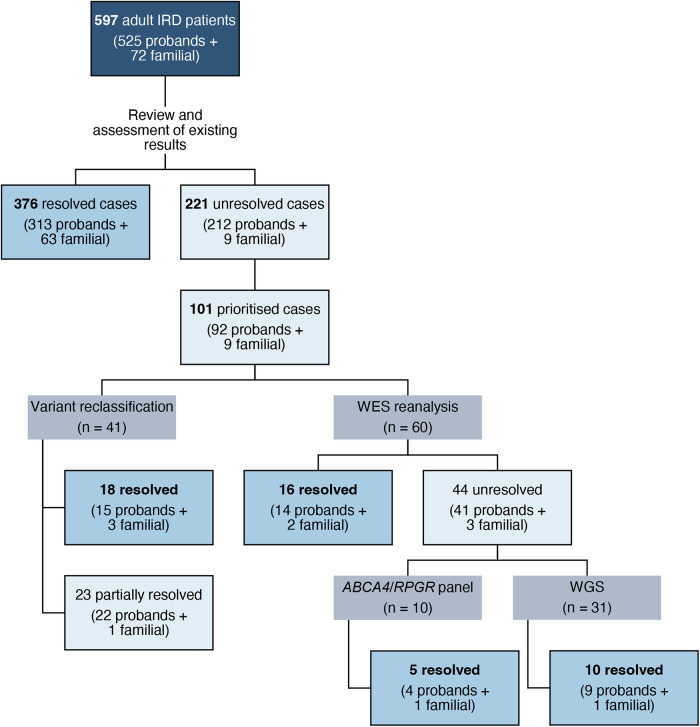
Diagnostic yield improvement through stepwise testing. Among the 101 unresolved cases prioritised for further assessment, 41 cases (37 probands and 4 familial) with phenotype-associated VUS underwent reclassification, leading to 18 new diagnoses (15 probands and 3 familial). The remaining 23 cases were considered partially resolved due to phenotype-matching VUS. WES reanalysis identified 16 additional diagnoses (14 probands and 2 familial), while targeted *ABCA4*/*RPGR* panel testing and WGS contributed to 5 and 10 new diagnoses, respectively. However, 29 cases (28 probands and 1 familial) remained inconclusive due to insufficient evidence of pathogenicity or genotype-phenotype discordance.

The 101 selected cases underwent further analysis through a stepwise, case-by-case strategy. Variant re-evaluation and reclassification were conducted for 41 cases with VUS that matched the clinical phenotype (P377–P417, Table 1), resolving 18 cases through VUS reclassification to likely pathogenic or pathogenic (Fig. 2). WES reanalysis identified 16 additional diagnoses, while WGS and customised gene panels provided molecular diagnoses for 15 more cases from a subset of 60 patients (P418–P477, Table 2).

**Table 1:** Cases with VUS and phenotype correlation.

ID	Age	Gender	AO	FamHx	Clinical diagnosis	Proband case	Final result	Gene	Transcript	Inh. pattern	Allele 1				Allele 2				Allele Segreg.
											cDNA	protein	variant ACMG classification	Criteria applied for VUS reassessment and reclassification	cDNA	protein	variant ACMG classification	Criteria applied for VUS reassessment and reclassification	
P377	62	M	50	Yes	Best macular dystrophy	Yes	Resolved	*BEST1*	NM_004183.4	AD	c.35C>A	p.(Ala12Asp)	LP	PM2_sup, PP3_mod, PM1_mod, PP2_sup					–
P378	67	F	5	Yes	Usher syndrome	Yes	Resolved	*CDH23*	NM_022124.6	AR	c.7199C>T	p.(Pro2400Leu)	LP	PM2_sup, PM3_strong, PP3_sup	c.7221C>A	p.(Tyr2407*)	P		*trans*
P379	25	F	21	No	Nonsyndromic retinitis pigmentosa	Yes	Resolved	*CNGA1*	NM_001379270.1	AR	c.1448T>G	p.(Leu483Arg)	LP	PM2_sup, PP3_strong, PM3_mod	c.82C>T	p.(Arg28*)	P		*trans*
P380	71	F	10	Yes	Nonsyndromic retinitis pigmentosa	Yes	Resolved	*CNGB1*	NM_001297.5	AR	c.1644-3C>G	p.(?)	LP	PM2_sup, PP3_sup, PM3_mod, PP1_strong	c.2868_2869insTA	p.(Val957*)	LP		*trans*
P381	52	F	21	Yes	Nonsyndromic retinitis pigmentosa	Yes	Resolved	*CNGB1*	NM_001297.5	AR	c.1644-3C>G	p.(?)	LP	PM2_sup, PP3_sup, PM3_mod, PP1_strong	c.2293C>T	p.(Arg765Cys)	LP		*trans*
P382	73	F	30	Yes	Stargardt disease	Yes	Resolved	*CRB1*	NM_201253.3	AR	c.4154A>C	p.(Glu1385Ala)	LP	PM2_sup,PM3_mod, PP1_mod, PP3_sup	c.498_506del	p.(Ile167_Gly169del)	P		*trans*
P383	70	F	35	Yes	Stargardt disease	No	Resolved	*CRB1*	NM_201253.3	AR	c.4154A>C	p.(Glu1385Ala)	LP	PM2_sup, PM3_mod, PP1_mod, PP3_sup	c.498_506del	p.(Ile167_Gly169del)	P		*trans*
P384	49	F	43	No	Maculopathy	Yes	Resolved	*CRB1*	NM_201253.3	AR	c.1760G>A	p.Cys587Tyr	LP	PM2_sup, PP3_sup, PM3_strong	c.613_619del	p.(Ile205Aspfs*13)	P		*trans*
P385	18	M	4	No	Cone-rod dystrophy	Yes	Resolved	*GUCY2D*	NM_000180.3	AD	c.543G>A	p.(Trp181*)	LP	PM2_sup, PVS1_very strong					-
P386	56	F	1	Yes	Leber congenital amaurosis	Yes	Resolved	*KCNJ13*	NM_002242.4	AR	c.859T>C	p.(Ser287Pro)	LP	PM2_sup, PP1_mod, PP3_strong	c.859T>C	p.(Ser287Pro)	LP		*trans*
P387	55	F	1	Yes	Leber congenital amaurosis	No	Resolved	*KCNJ13*	NM_002242.4	AR	c.859T>C	p.(Ser287Pro)	LP	PM2_sup, PP1_mod, PP3_strong	c.859T>C	p.(Ser287Pro)	LP		*trans*
P388	39	F	5	No	Albinism	Yes	Resolved	*OCA2*/*TYR*	NM_000275.2/NM_000372.4	digenic	c.1327G>A	p.(Val443Ile)	P	PM3_very strong, PP4_mod, PS3_sup	c.1205G>A	p.(Arg402Gln)	Polymorphism		Not available
P389	54	F	33	Yes	Nonsyndromic retinitis pigmentosa	Yes	Resolved	*PCARE*	NM_001029883.3	AR	c.2677C>T	p.(Pro893Ser)	LP	PM2_sup, PM3_strong, PP1_mod	c.2950C>T	p.(Arg984*)	P		*trans*
P390	48	F	14	Yes	Nonsyndromic retinitis pigmentosa	No	Resolved	*PCARE*	NM_001029883.3	AR	c.2677C>T	p.(Pro893Ser)	LP	PM2_sup, PM3_strong, PP1_mod	c.2950C>T	p.(Arg984*)	P		*trans*
P391	73	F	35	Yes	Nonsyndromic retinitis pigmentosa	Yes	Resolved	*PDE6A*	NM_000440.2	AR	c.1065+1G>T	p.(?)	P	PM2_sup, PVS1, PM3_mod	c.1957C>T	p.(Arg653*)	P		*trans*
P392	56	M	50	No	Nonsyndromic retinitis pigmentosa	Yes	Resolved	*POMGNT1*	NM_017739.4	AR	c.629G>T	p.(Trp210Leu)	LP	PM2_sup, PP3_mod, PM5_mod, PM3_sup	c.860T>G	p.(Ile287Ser)	P		*trans*
P393	59	M	55	Yes	Maculopathy	Yes	Resolved	*RDH5*	NM_002905.5	AD	c.592del	p.(Ile198Tyrfs*15)	LP	PM2_sup, PVS1_very strong					-
P394	59	M	22	Yes	Nonsyndromic retinitis pigmentosa	Yes	Resolved	*REEP6*	NM_138393.4	AR	c.349-4G>T	p.(Cys117Argfs*59)	LP	PM2_sup, PVS1_very strong	c.349-1G>A	p.(Cys117Argfs*59)	P		*trans*
P395	52	F	1	No	Leber congenital amaurosis	Yes	Partially resolved	*AIPL1*	NM_014336.4	AR	c.767T>G	p.(Ile256Ser)	VUS	PM2_sup, PP3_sup, PM3_mod	c.767T>G	p.(Ile256Ser)	VUS	PM2_sup, PP3_sup, PM3_mod	*trans*
P396	59	M	50	No	Best macular dystrophy	Yes	Partially resolved	*BEST1*	NM_004183.4	AD	c.847T>C	p.(Phe283Leu)	VUS	PM2_sup, PP3_mod, PM1_mod					ND
P397	32	M	5	No	Usher syndrome	Yes	Partially resolved	*CDH23*	NM_022124.6	AR	c.8699A>G	p.(Asp2900Gly)	VUS	PM2_sup, BP4_sup, PM3_mod	c.289-1_304del	p.(?)	LP		*trans*
P398	57	M	45	No	Cone-rod dystrophy	Yes	Partially resolved	*CDHR1*	NM_033100.4	AR	c.151G>A	p.(Gly51Ser)	VUS	PM2_sup, PP3_sup, PM3_mod	c.2522_2528del	p.(Ile841Serfs*119)	LP		*trans*
P399	87	F	50	No	Cone-rod dystrophy	Yes	Partially resolved	*CDHR1*	NM_033100.4	AR	c.1367C>A	p.(Ala456Glu)	VUS	PM2_sup, PP3_strong	c.1485+2T>G	p.(?)	P		*trans*
P400	52	F	10	No	Cone-rod dystrophy	Yes	Partially resolved	*CDHR1*	NM_033100.4	AR	c.1367C>A	p.(Ala456Glu)	VUS	PM2_sup, PP3_strong	c.1367C>A	p.(Ala456Glu)	VUS	PM2_sup, PP3_strong	*trans*
P401	77	F	40	No	Cone-rod dystrophy	Yes	Partially resolved	*CFAP410*	NM_004928.2	AR	c.140T>C	p.(Leu47Pro)	VUS	PM2_sup	c.140T>C	p.(Leu47Pro)	VUS	PM2_sup	*trans*
P402	79	F	20	No	Nonsyndromic retinitis pigmentosa	Yes	Partially resolved	*CNGB1*	NM_001297.5	AR	c.2095G>A	p.(Asp699Asn)	VUS	PM2_sup	c.2095G>A	p.(Asp699Asn)	VUS	PM2_sup	*trans*
P403	71	M	65	No	Nonsyndromic retinitis pigmentosa	Yes	Partially resolved	*EYS*	NM_001142800.2	AR	c.8627G>T	p.(Gly2876Val)	VUS	PM2_sup	c.8143C>T	p.(Arg2715*)	P		*trans*
P404	84	M	50	No	Nonsyndromic retinitis pigmentosa	Yes	Partially resolved	*FSCN2*	NM_001077182.3	AD	c.1444del	p.(Arg482Alafs)	VUS	PM2_sup, PM4_mod					-
P405	47	M	10	No	Leber congenital amaurosis	Yes	Partially resolved	*GUCY2D*	NM_000180.4	AR	c.736A>C	p.(Met246Leu)	VUS	PM2_sup, PM1_mod, PM3_sup	c.3025A>C	p.(Met1009Leu)	P		*trans*
P406	26	M	20	No	Familial exudative vitreoretinopathy	Yes	Partially resolved	*LRP5*	NM_002335.4	AD	c.3220G>T	p.(Val1074Phe)	VUS	PM2_sup, PP3_mod, PP1_sup					ND
P407	20	M	15	Yes	Familial exudative vitreoretinopathy	No	Partially resolved	*LRP5*	NM_002335.4	AD	c.3220G>T	p.(Val1074Phe)	VUS	PM2_sup, PP3_mod, PP1_sup					ND
P408	63	M	40	Yes	Nonsyndromic retinitis pigmentosa	Yes	Partially resolved	*MERTK*	NM_006343.3	AR	c.950G>A	p.(Cys317Tyr)	VUS	PM2_sup	c.2215_2216del	p.(Val739Cysfs*7)	P		*trans*
P409	34	M	10	No	Usher syndrome	Yes	Partially resolved	*MYO7A*	NM_000260.4	AR	c.5537C>A	p.(Pro1846His)	VUS	PM2_sup, PP3_sup, PM3_mod	c.6025del	p.(Ala2009Profs*32)	P		*trans*
P410	72	F	unknown	No	Nonsyndromic retinitis pigmentosa	Yes	Partially resolved	*PDE6B*	NM_000283.4	AR	c.1148T>A	p.(Val383Glu)	VUS	PM2_sup, PP3_mod, PM3_sup	c.299G>A	p.(Arg100His)	LP		*trans*
P411	50	F	4	No	Nonsyndromic retinitis pigmentosa	Yes	Partially resolved	*PDE6B*	NM_000283.4	AR	c.2176T>G	p.(Trp726Gly)	VUS	PM2_sup, PP3_sup	c.1879C>G	p.(Arg627Gly)	VUS	PM2_sup, PP3_sup	*trans*
P412	25	M	3	No	Achromatopsia	Yes	Partially resolved	*PDE6C*	NM_006204.4	AR	c.1950C>A	p.(Phe650Leu)	VUS	PM2_sup, PP3_sup	c.712C>T	p.(Arg238*)	P		*trans*
P413	46	F	16	No	Fundus albipunctatus	Yes	Partially resolved	*RDH5*	NM_002905.5	AD/AR	c.463C>T	p.(Arg155Trp)	VUS	PM2_sup, PP4_sup	c.779A>C	p.(Asp260Ala)	VUS	PM1_sup, PM2_sup, PP3_sup, PP4_sup	ND
P414	40	F	17	Yes	Nonsyndromic retinitis pigmentosa	Yes	Partially resolved	*USH2A*	NM_206933.4	AR	c.12403T>C	p.(Cys4135Arg)	VUS	PM2_sup, PM3_sup	c.2276G>T	p.(Cys759Phe)	P		ND
P415	88	F	70	No	Nonsyndromic retinitis pigmentosa	Yes	Partially resolved	*USH2A*	NM_206933.4	AR	c.7951A>G	p.(Asn2651Asp)	VUS	PM2_sup, BP4_sup, PM3_mod	c.2276G>T	p.(Cys759Phe)	P		*trans*
P416	67	F	35	Yes	Usher syndrome	Yes	Partially resolved	*USH2A*	NM_206933.4	AR	c.1106T>A	p.(Val369Glu)	VUS	PM2_sup, PP3_sup, PS4_sup, PM3_sup	c.12575G>A	p.(Arg4192His)	LP		*trans*
P417	62	M	58	No	Nonsyndromic retinitis pigmentosa	Yes	Partially resolved	*ZNF408*	NM_024741.3	AR	c.1342C>T	p.(Arg448Cys)	VUS	PM2_sup, BP4_mod, PM3_mod	c.1342C>T	p.(Arg448Cys)	VUS	PM2_sup, BP4_mod, PM3_mod	ND

*AD* autosomal dominant, *AO* age of onset, *AR* autosomal recessive, *FamHx* family history of IRDs, *F* female, *LP* likely pathogenic, *M* male, *mod* moderate, *ND* not determined, *P* pathogenic, *sup* supporting, *VUS* variant of uncertain significance.

**Table 2:** Additional genomic analyses and results in unresolved cases.

ID	Age	Gender	AO	FamHx	Clinical diagnosis	Proband case	Approach	Final result	Gene	Transcript	Inh. pattern	Allele 1		Allele 2		Phenotypic match?	Segregation
												cDNA	protein	cDNA	protein		
P418	54	M	20	Yes	Nonsyndromic retinitis pigmentosa	Yes	WES reanalysis + Digital PCR	Resolved	*ARSG*	NM_001267727.2	AR	**Chr17:66246319-66303862del**	p.(?)	**c.983-2_983-1del**	p.(?)	Yes	*trans*
P419	51	F	48	Yes	Nonsyndromic retinitis pigmentosa	No	Sanger Sequencing + Digital PCR	Resolved	*ARSG*	NM_001267727.2	AR	**Chr17:66246319-66303862del**	p.(?)	**c.983-2_983-1del**	p.(?)	Yes	*trans*
P420	24	F	12	No	Stargardt disease	Yes	WES reanalysis + *ABCA4* panel	Resolved	*ABCA4*	NM_000350.3	AR	c.634C>T	p.(Arg212Cys)	**c.4539+2064C**>**T**	p.[=,Arg1514Leufs*36]	Yes	*trans*
P421	52	M	41	No	Stargardt disease	Yes	WES reanalysis	Resolved	*ABCA4*	NM_000350.3	AR	c.[3210_3211dup;5603A>T]	p.[Ser1071Cfs*14;Asn1868Ile]	**c.5603A**>**T**	p.(Asn1868Ile)	Yes	*trans*
P422	40	F	25	No	Stargardt disease	Yes	WES reanalysis	Resolved	*ABCA4*	NM_000350.3	AR	c.6056A>T	p.(Gln2019Leu)	**c.1166C**>**A**	p.(Ala389Glu)	Yes	*trans*
P423	51	M	48	No	Late onset Stargardt disease	Yes	WES reanalysis + *ABCA4* panel	Resolved	*ABCA4*	NM_000350.3	AR	c.[5044_5058del;4926C>G]	p.[Val1682_Val1686del;Ser1642Arg]	**c.5196+1137G**>**A**	p.[=,Met1733Glufs*78]	Yes	*trans*
P424	27	F	14	No	Stargardt disease	Yes	WES reanalysis	Resolved	*ABCA4*	NM_000350.3	AR	c.5882G>A	p.(Gly1961Glu)	**c.4877C**>**A**	p.(Ala1626Asp)	Yes	*trans*
P425	79	F	56	No	Late onset Stargardt disease	Yes	WES reanalysis	Resolved	*ABCA4*	NM_000350.3	AR	c.3386G>T	p.(Arg1129Leu)	**c.1244A**>**G**	p.(Asn415Ser)	Yes	*trans*
P426	53	F	50	No	Late onset Stargardt disease	Yes	WES reanalysis	Resolved	*ABCA4*	NM_000350.3	AR	c.6272T>G	p.(Leu2091Arg)	**c.5603A**>**T**	p.(Asn1868Ile)	Yes	*trans*
P427	30	M	27	Yes	Stargardt disease	Yes	WES reanalysis	Resolved	*ABCA4*	NM_000350.3	AR	c.3386G>T	p.(Arg1129Leu)	**c.52C**>**T**	p.(Arg18Trp)	Yes	*trans*
P428	55	M	50	No	Late onset Stargardt disease	Yes	WES reanalysis + WGS	Resolved	*ABCA4*	NM_000350.3	AR	**c.3056C**>**T**	p.(Thr1019Met)	c.4253+43G>A	p.[=,Ile1377Hisfs*3]	Yes	*trans*
P429	70	F	55	No	Late onset Stargardt disease	Yes	WES reanalysis + WGS	Resolved	*ABCA4*	NM_000350.3	AR	c.3386G>T	p.(Arg1129Leu)	**c.4539+2064C**>**T**	p.[=,Arg1514Leufs*36]	Yes	*trans*
P430	58	F	40	No	Stargardt disease	Yes	WES reanalysis + WGS	Resolved	*ABCA4*	NM_000350.3	AR	**c.5196+1137G**>**A**	p.[=,Met1733Glufs*78]	c.[5461-10T>C;5603A>T]	p.[Thr1821Aspfs*6,Thr1821Valfs*13;Asn1868Ile]	Yes	*trans*
P431	44	M	35	No	Stargardt disease	Yes	WES reanalysis	Resolved	*ABCA4*	NM_000350.3	AR	c.[3617del;5603A>T]	p.[Asn1206Metfs*3;Asn1868Ile]	**c.5603A**>**T**	p.(Asn1868Ile)	Yes	*trans*
P432	59	M	40	No	Stargardt disease	Yes	WES reanalysis + WGS	Resolved	*ABCA4*	NM_000350.3	AR	c.3386G>T	p.(Arg1129Leu)	**c.4539+2064C**>**T**	p.[=,Arg1514Leufs*36]	Yes	*trans*
P433	59	M	54	No	Late onset Stargardt disease	Yes	WES reanalysis + WGS + MLPA	Resolved	*ABCA4*	NM_000350.3	AR	**c.699_768+341del**	p.(Gln234Phefs*5)	**c.5603A**>**T**	p.(Asn1868Ile)	Yes	*trans*
P434	30	F	5	No	Stargardt disease	Yes	WES reanalysis + *ABCA4* panel + WGS + Midigene	Resolved	*ABCA4*	NM_000350.3	AR	c.634C>T	p.(Arg212Cys)	**c.859-442C**>**T**	p.[Phe287Tyrfs*33,Phe287_Glu518del,=, Phe287Hisfs*7]	Yes	*trans*
P435	55	M	15	Yes	Syndromic retinitis pigmentosa	Yes	WES reanalysis	Resolved	*NPHP4*	NM_015102.5	AR	**c.2485+2T**>**C**	p.(?)	**c.2611+1G**>**A**	p.(?)	Yes	*trans*
P436	51	M	36	Yes	Syndromic retinitis pigmentosa	No	Sanger Sequencing	Resolved	*NPHP4*	NM_015102.5	AR	**c.2485+2T**>**C**	p.(?)	**c.2611+1G**>**A**	p.(?)	Yes	*trans*
P437	54	M	20	No	Cone-rod dystrophy	Yes	WES reanalysis + Minigen	Resolved	*TULP1*	NM_003322.6	AR	**c.822G**>**T**	p.(Lys274Asn)	c.1376T>C	p.(Ile459Thr)	Yes	*trans*
P438	60	M	31	Yes	Nonsyndromic retinitis pigmentosa	Yes	WES reanalysis + *RPGR* panel	Resolved	*RPGR*	NM_001034853.2	XL	**c.3116dup**	p.(Glu1040Argfs*39)			Yes	inherited
P439	46	M	28	Yes	Nonsyndromic retinitis pigmentosa	No	Sanger Sequencing	Resolved	*RPGR*	NM_001034853.2	XL	**c.3116dup**	p.(Glu1040Argfs*39)			Yes	inherited
P440	51	F	30	No	Syndromic retinitis pigmentosa	Yes	WES reanalysis + WGS + aCGH	Resolved	*NPHP1*	NM_001128178.3	AR	**chr2:110853694-110985405del**	p.(?)	**chr2:110853694-110985405del**	p.(?)	Yes	*trans*
P441	33	F	25	Yes	Nonsyndromic retinitis pigmentosa	Yes	WES reanalysis + WGS	Resolved	*CERKL*	NM_201548.5	AR	**c.769C**>**T**	p.(Arg257*)	**c.769C**>**T**	p.(Arg257*)	Yes	*trans*
P442	24	M	6	No	Nonsyndromic retinitis pigmentosa	Yes	WES reanalysis + *RPGR* panel	Resolved	*RPGR*	NM_001034853.2	XL	**c.2527del**	p.(Glu843Lysfs*246)			Yes	inherited
P443	37	F	10	No	Nonsyndromic retinitis pigmentosa	Yes	WES reanalysis + MLPA	Resolved	*PDE6B*	NM_000283.4	AR	**c.(?_-53)_(711** + **1_712-1)del**	p.(?)	**c.(?_-53)_(711** + **1_712-1)del**	p.(?)	Yes	*trans*
P444	22	M	4	No	Syndromic retinitis pigmentosa	Yes	WES reanalysis	Resolved	*HK1*	NM_033500.2	AD	**c.1334C**>**G**	p.(Thr445Arg)			Yes	de novo
P445	19	M	5	No	Cone-rod dystrophy	Yes	WES reanalysis	Resolved	*RPGRIP1*	NM_020366.3	AR	c.1111C>T	p.(Arg371*)	**c.2367+23delG**	p.(?)	Yes	*trans*
P446	27	F	2	Yes	Usher syndrome	Yes	WES reanalysis + WGS	Resolved	*USH2A*	NM_206933.4	AR	c.2299del	p.(Glu767Serfs*21)	**c.11048-1055A**>**G**	p.(?)	Yes	*trans*
P447	30	M	16	Yes	Usher syndrome	No	Sanger Sequencing	Resolved	*USH2A*	NM_206933.4	AR	c.2299del	p.(Glu767Serfs*21)	**c.11048-1055A**>**G**	p.(?)	Yes	*trans*
P448	21	F	10	No	Cone dystrophy	Yes	WES reanalysis + mRNA	Resolved	*ATF6*	NM_007348.4	AR	**c.160-8A**>**G**	p.(Glu54Phefs*7)	**c.160-8A**>**G**	p.(Glu54Phefs*7)	Yes	*trans*
P449	46	M	20	Yes	Syndromic retinitis pigmentosa	Yes	WES reanalysis + *ABCA4* panel	Inconclusive	*ABCA4*	NM_000350.3	AR	c.6424A>G	p.(Ile2142Val)	c.5603A>T	p.(Asn1868Ile)	Yes	*trans*
P450	59	M	56	Yes	Retinal pattern dystrophy	Yes	WES reanalysis + *ABCA4* panel	Inconclusive	*ABCA4*	NM_000350.3	AR	c.5908C>T	p.(Leu1970Phe)	c.2744-9del	p.(?)	Yes	*trans*
P451	78	F	50	Yes	Late onset Stargardt disease	Yes	WES reanalysis + WGS	Inconclusive	*ABCA4*	NM_000350.3	AR	c.5882G>A	p.(Gly1961Glu)			Yes	ND
P452	59	F	50	No	Nonsyndromic retinitis pigmentosa	Yes	WES reanalysis + *ABCA4* panel	Inconclusive	*ABCA4*	NM_000350.3	AR	c.3113C>T	p.(Ala1038Val)			Yes	ND
P453	39	M	20	No	Maculopathy	Yes	WES reanalysis + *ABCA4* panel	Inconclusive	*ABCA4*	NM_000350.3	AR	c.4457C>T	p.(Pro1486Leu)			Yes	ND
P454	78	F	30	Yes	Nonsyndromic retinitis pigmentosa	Yes	WES reanalysis	Inconclusive	*AHI1*	NM_017651.5	AR	c.1205del	p.(Pro402Leufs*3)			No	ND
P455	59	M	40	Yes	Cone-rod dystrophy	Yes	WES reanalysis + WGS	Inconclusive	*AIPL1*	NM_014336.5	AD	c.112C>T	p.(Arg38Cys)			Yes	ND
P456	63	M	38	Yes	Cone-rod dystrophy	No	Sanger Sequencing	Inconclusive	*AIPL1*	NM_014336.5	AD	c.112C>T	p.(Arg38Cys)			Yes	ND
P457	56	F	17	No	Nonsyndromic retinitis pigmentosa	Yes	WES reanalysis + WGS	Inconclusive								N/A	ND
P458	67	M	47	No	Cone-rod dystrophy	Yes	WES reanalysis + WGS	Inconclusive	*SPG7*	NM_003119.4	AR	c.1529C>T	p.(Ala510Val)			No	ND
P459	70	M	12	No	Nonsyndromic retinitis pigmentosa	Yes	WES reanalysis + WGS	Inconclusive	*ABCA4*	NM_000350.3	AR	c.5603A>T	p.(Asn1868Ile)	c.3607+771G>A	p.(=)	Yes	ND
P460	62	M	55	Yes	Late onset Stargardt disease	Yes	WES reanalysis + WGS	Inconclusive								No	ND
P461	36	F	27	No	Nonsyndromic retinitis pigmentosa	Yes	WES reanalysis + WGS	Inconclusive	*MYO3A*	NM_017433.5	AR	c.4550C>G	p.(Ser1517*)			No	ND
P462	33	F	20	No	Nonsyndromic retinitis pigmentosa	Yes	WES reanalysis + WGS	Inconclusive	*RDH12*	NM_152443.3	AD/AR	c.464C>T	p.(Thr155Ile)			Yes	inherited
P463	57	M	52	Yes	Nonsyndromic retinitis pigmentosa	Yes	WES reanalysis + WGS	Inconclusive	*CNGA1*	NM_001379270.1	AR	c.349G>T	p.(Glu117*)			Yes	ND
P464	47	M	35	No	Nonsyndromic retinitis pigmentosa	Yes	WES reanalysis + WGS	Inconclusive	*ABCA4*	NM_000350.3	AR	c.5843C>T	p.(Pro1948Leu)			Yes	ND
P465	56	F	24	Yes	Central areolar choroidal dystrophy	Yes	WES reanalysis + *ABCA4* panel + WGS	Inconclusive	*ABCA4*	NM_000350.3	AR	c.[5603A>T;2701A>G]	p.[Asn1868Ile;Thr901Ala]			Yes	ND
P466	60	M	52	No	Maculopathy	Yes	WES reanalysis + WGS	Inconclusive	*ABCA4*	NM_000350.3	AR	c.6148G>C	p.(Val2050Leu)			Yes	ND
P467	27	F	23	No	Nonsyndromic retinitis pigmentosa	Yes	WES reanalysis + WGS	Inconclusive								N/A	ND
P468	65	F	55	No	Retinal pattern dystrophy	Yes	WES reanalysis + *ABCA4* panel	Inconclusive	*ABCA4*	NM_000350.3	AR	c.4919G>A	p.(Arg1640Gln)			Yes	ND
P469	70	F	55	Yes	Late onset Stargardt disease	Yes	WES reanalysis + *ABCA4* panel + WGS	Inconclusive	*ABCA4*	NM_000350.3	AR	c.[5549T>C;5603A>T]	p.[Leu1850Pro;Asn1868Ile]			Yes	ND
P470	50	F	2	No	Nonsyndromic retinitis pigmentosa	Yes	WES reanalysis + WGS	Inconclusive	*MKS1*	NM_017777.4	AR	c.190+2T>C	p.(?)			No	ND
P471	71	F	67	No	Retinal pattern dystrophy	Yes	WES reanalysis	Inconclusive	*NRL*	NM_001354768.3	AD/AR	c.654del	p.(Cys219Valfs*4)			No	ND
P472	67	F	42	No	Nonsyndromic retinitis pigmentosa	Yes	WES reanalysis + WGS	Inconclusive	*ABCA4*	NM_000350.3	AR	c.5603A>T	p.(Asn1868Ile)	c.4539+859C>T	p.(=)	Yes	ND
P473	48	M	11	No	Maculopathy	Yes	WES reanalysis + WGS	Inconclusive								N/A	ND
P474	51	F	46	No	Nonsyndromic retinitis pigmentosa	Yes	WES reanalysis + WGS + digital PCR	Inconclusive	*ARHGEF18*	NM_001367823.1	AR	Chr19:7434598-7442499-del	p.(?)			Yes	ND
P475	50	F	43	Yes	Retinal pattern dystrophy	Yes	WES reanalysis + WGS	Inconclusive								N/A	ND
P476	41	F	28	No	Nonsyndromic retinitis pigmentosa	Yes	WES reanalysis + WGS	Inconclusive								N/A	ND
P477	38	F	15	No	Cone-rod dystrophy	Yes	WES reanalysis	Inconclusive	*VPS13B*	NM_017890.5	AR	c.11598del	p.(Glu3867Lysfs*11)			Yes	ND

*AD* autosomal dominant, *AO* age of onset, *AR* autosomal recessive, *FamHx* family history of IRDs, *F* female, *M* male, *ND* not determined, *XL* X-linked inheritance.

Variants identified through the re-evaluation are highlighted in bold.

In total, 49 new molecular diagnoses were established, comprising 42 probands and 7 familial cases. This personalised approach achieved a diagnostic rate of 48.5% (49/101) in reassessed cases and increased the overall diagnostic rate for probands to 67.6% (355/525), reflecting a 13.4% relative improvement in diagnostic yield.

### Reclassification of candidate variants

Family co-segregation and functional studies played crucial roles in reclassifying VUS. In patient P394, the *REEP6* c.349-4G>T variant, initially classified as a VUS, was upgraded to likely pathogenic following co-segregation and mRNA analysis, which revealed a 32 nt deletion in exon 4 resulting in a frameshift (Supplementary Fig. S1). Computational predictions (SpliceAI) predicted minimal splicing impact (acceptor loss: 0.07; cryptic acceptor activation: 0.16) (Supplementary Table S4), but cDNA sequencing demonstrated loss-of-function, supporting pathogenicity. Conversely, despite a strong genotype-phenotype correlation, the *AIPL1* c.767T>G variant identified in case P395 remained classified as a VUS due to insufficient functional evidence.

In addition to the 18 resolved cases, the reclassification of variants also contributed to the partial resolution of 23 additional cases, for which future evidence may provide further insights leading to conclusive classification.

### Non-coding variants

Pathogenic non-coding variants were identified in *ABCA4*, *ATF6*, *NPHP4*, *RPGRIP1* and *USH2A* genes through WES reanalysis, a customised *ABCA4* panel and WGS (Table 2). Seven deep intronic variants in *ABCA4* were detected, including six previously reported [36] (c.4539+2064C>T in P420, P429 and P432; c.5196+1137G>A in P423 and P430; and c.4253+43G>A in P428) and a novel c.859-442C>T variant in patient P434. Segregation analysis clarified cases initially classified as resolved. For example, in patients P423 and P430, it confirmed that *ABCA4* variants were in *cis*, leading to the identification of deep intronic variants in *trans*, which resolved both cases.

**Fig. 3:**
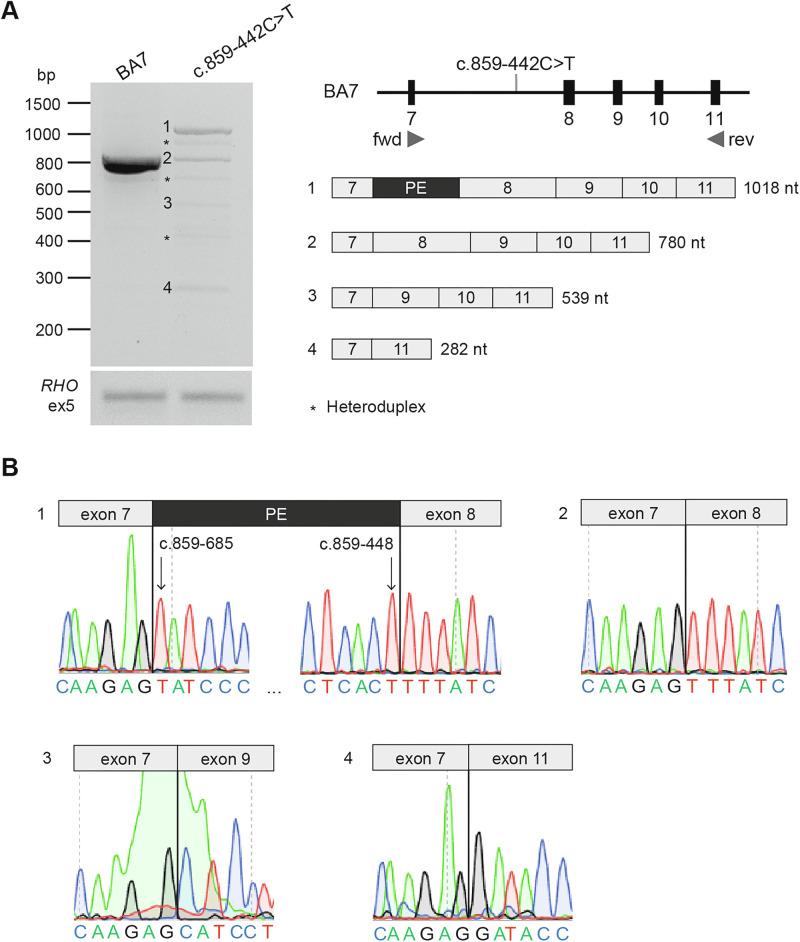
Overview of splice defects caused by *ABCA4* c.859-442C>T variant in HEK293T cells. **A** Wild-type and mutant midigenes assay results. *Rhodopsin* exon 5 (*RHO* ex5) RT-PCR was used as a control for transfection efficiency. To the right, schematic representation of WT midigene (BA7_WT), in which the position of the variant is indicated with an arrow and the forward (fwd) and reverse (rev) primers used for PCR amplification are depicted as triangles. Beneath, schematic representation of the four RT-PCR products identified in panel, heteroduplex bands are labelled with an asterisk. *ABCA4* c.859-442C>T variant leads to the inclusion of a 238 nt long pseudoexon (PE) in intron 7 (Fragment 1), exon 8 skipping (Fragment 3), exon 8 to 10 skipping (Fragment 4) and WT product (Fragment 2). **B** The chromatograms show the exact exonic and intronic breakpoints in the four fragments as confirmed by Sanger sequencing.

WGS identified the novel *ABCA4* c.859-442C>T variant in P434. SpliceAI predictions indicated acceptor and donor gain (acceptor gain: 0.28; donor gain: 0.23) (Supplementary Table S4). In vitro splice assays revealed three splicing alterations: inclusion of a 238 nt pseudoexon (37%), exons 8–10 skipping (35%) and exon 8 skipping (7%) (Supplementary Table S5), which resulted in frameshifts and premature stop codons, likely disrupting *ABCA4* function (Fig. 3). Consequently, the c.859-442C>T variant was classified as moderately-severe in line with previous severity *ABCA4* variants classifications [28, 38, 41].

In patient P448, diagnosed with non-progressive cone-rod dystrophy at age 10, WES reanalysis identified a homozygous *ATF6* c.160-8A>G variant with a strong genotype-phenotype correlation. SpliceAI predicted a highly impactful acceptor gain (score: 0.99) and a minor acceptor loss (score 0.11) (Supplementary Table S4). mRNA analysis confirmed the inclusion of 7 nt of intron 2 into de coding sequence, leading to a frameshift and introducing a premature stop codon (Supplementary Fig. S2). This led to the reclassification of the variant as likely pathogenic.

In siblings P435 and P436, biallelic splice-site *NPHP4* variants (c.2485+2T>C and c.2611+1G>A) were identified, which had been missed due to the absence of *NPHP4* from the original virtual panel. This finding confirmed the diagnosis of Senior-Løken syndrome and revealed previously unrecognised kidney involvement in one sibling.

WES reanalysis also identified a second *RPGRIP1* c.2367+23del variant in *trans* with a previously detected c.1111C>T pathogenic variant in patient P445, confirming the molecular diagnosis. Additionally, WGS identified a novel deep intronic *USH2A* c.11048-1055A>G variant in siblings P446 and P447, reinforcing their clinical diagnosis.

### Structural and copy number variants

WGS detected previously overlooked SVs (Table 2), including a partial exon 6 deletion in *ABCA4* in patient P433 and a homozygous deletion affecting three genes, including *NPHP1*, in patient P440, who presented with retinitis pigmentosa and renal disease. WES reanalysis identified a homozygous intragenic *PDE6B* deletion in P443 and a likely pathogenic deletion involving *ARSG* exon 2 in P418, which had been missed due to limitations in the original analysis pipeline for detecting CNVs.

### Coding variants and emerging gene associations

WES reanalysis identified previously overlooked coding variants in *ABCA4*, *CERKL*, *HK1*, *RPGR*, and *TULP1* (Table 2). Emerging functional evidence, newly reported gene-disease associations, and advances in bioinformatics facilitated the detection and reclassification of variants. For example, biallelic *ABCA4* variants, including the c.5603A>T hypomorphic variant, were identified in several cases (P421, P422, P424, P425, P426, P427 and P431). Additionally, a pathogenic homozygous *CERKL* c.769C>T variant was detected in patient P441, previously missed due to outdated transcript annotation.

In patient P444, a de novo pathogenic *HK1* c.1334C>G variant was identified through WES reanalysis, guided by HPO terms. In patients P438, P439 and P442, variants in *RPGR*-ORF15 were detected using a customised *RPGR* panel, which facilitated the detection of variants in low-coverage regions. Finally, a novel splice-site *TULP1* c.822G>T variant was identified in patient P437, with its pathogenicity validated through a minigene assay previously reported by our group [39].

## Discussion

This study demonstrates a significant improvement in the molecular diagnostic yield for IRDs through a patient-centred, multi-step genomic approach. By integrating variant reclassification, WES reanalysis, WGS, customised gene panels and functional assays, we resolved previously undiagnosed cases, providing deeper insights into IRD pathogenesis. These findings highlight the diagnostic challenges posed by the genetic heterogeneity of IRDs, particularly the presence of SVs and variants in GC-rich and non-coding regions, which are often missed by conventional methods [42].

The initial WES diagnostic yield in our cohort was 59.6%, aligning with previously reported rates for IRDs [3, 12–14]. However, incorporating variant reclassification, additional sequencing and functional validation increased the yield to 48.5% among the prioritised cases that were re-evaluated. This surpasses diagnostic rates reported in large cohort studies where WGS alone was used as a second-tier method (33.3% [19], 24% [27] and 13% [18]), emphasising the value of a personalised approach.

While WES and WGS significantly contributed to variant identification, the interpretation of VUS remains challenging. Cases P394 and P448 exemplify how segregation analysis and functional assays can refine variant classification and resolve previously inconclusive cases. In contrast, patient P395 remained unresolved despite a strong genotype-phenotype correlation, underscoring the need for periodic reassessment and functional validation beyond in silico predictions.

The *ABCA4* c.5603A>T hypomorphic variant, now recognised as pathogenic [22], was initially not reported due to limited evidence of pathogenicity. This variant is estimated to account for approximately 50% of unresolved cases in individuals carrying only one *ABCA4* pathogenic variant [43], reinforcing the need for WES reanalysis as new evidence emerges [44]. However, its interpretation requires caution, as it is only considered pathogenic when in *trans* with severe variants. For instance, in cases P449, P459 and P472, c.5603A>T was found in *trans* with non-loss-of-function variants, limiting resolution of these cases.

Additionally, updates to transcript annotation were crucial, as demonstrated by case P441, where a *CERKL* variant was initially undetected. Similarly, in other cases, the detection of causative variants was hindered by the absence of certain genes in the applied virtual panels, highlighting the need for continuous updates to gene lists, transcript-aware analysis and periodic WES reanalysis using updated bioinformatics pipelines [44, 45].

One case was resolved through HPO-driven reanalysis, demonstrating its utility in syndromic cases, though its impact on non-syndromic IRDs remains limited [46]. Furthermore, the use of a customised *RPGR* panel enriched for low-coverage regions proved particularly effective in detecting variants within the ORF15 region, providing a cost-effective alternative to WGS and long-read sequencing technologies for sequencing this hotspot [47, 5].

Our findings reinforce the role of non-coding variants in IRD pathogenesis [18]. The identification of pathogenic intronic variants in *ABCA4*, *ATF6*, *NPHP4*, *RPGRIP1* and *USH2A* further validate their significance in disease development [4, 25, 48]. Notable examples include the novel deep intronic variants *ABCA4* c.859-442C>T and *USH2A* c.11048-1055A>G, both classified as pathogenic following segregation and/or functional analyses. The acceptor gain position at *ABCA4* c.859-685 (243 nt upstream of -442) appears to be recurrently activated, as shown in Khan et al. (2020) and Corradi et al. (2022) [49, 50], where it coincided with pseudo-exon inclusion and the largest exon elongation for the -25A>G variant of intron 7. Another variant using this splice acceptor site has also been reported [22], reinforcing its functional relevance. These findings highlight the need for routine non-coding region screening, particularly in *ABCA4* and *USH2A*. Cases P423 and P430 further emphasise the importance of segregation analysis to prevent misinterpretation when pathogenic variants are inherited in *cis*.

The identification of SVs—including a previously overlooked homozygous intragenic *PDE6B* deletion, a partial exon 6 deletion in *ABCA4*, which represents the second most frequently reported SV in *ABCA4*, particularly prevalent in the Spanish population [51] and a large deletion involving *NPHP1*—, reinforces the need to integrate WGS as a second-tier test in unresolved cases. These findings highlight the limitations of WES in detecting complex genomic rearrangements and emphasise the need for complementary approaches to improve diagnostic accuracy [18, 19].

Beyond diagnostics, these findings have direct clinical implications. Establishing a molecular diagnosis enables tailored genetic counselling, informed clinical decision-making and eligibility for emerging gene-specific therapies [52]. In addition to the 49 new diagnoses, segregation analysis in asymptomatic individuals identified carriers, facilitating reproductive counselling in at-risk couples, some of whom opted for preimplantation genetic diagnosis, directly impacting the next generation. Moreover, the identification of previously undetected variants enhances our understanding of disease mechanisms, which is crucial for developing more precise molecular diagnostic protocols for IRDs [41, 53].

Based on our findings, we propose a flexible and scalable diagnostic workflow for IRDs that integrates reanalysis, WGS and functional assays as complementary tools. Targeted gene panels and WES remain cost-effective and reliable first-line options in many healthcare settings, especially when their design is periodically updated to include newly associated IRD genes. However, clinicians must be aware of their limitations in detecting non-coding, structural and complex variants. Current literature supports systematic reanalysis of WES data every 18 to 24 months, due to ongoing advances in gene discovery and variant interpretation [54]. Nonetheless, timing should remain flexible and adapted to individual clinical contexts, particularly when preliminary findings suggest the presence of deep intronic or SVs, or when reproductive planning is a priority [16]. In selected cases with strong genotype–phenotype correlation, such as individuals carrying a single pathogenic variant in *ABCA4* or *USH2A*, re-evaluation should not be delayed, even if the initial study was recent, as deep intronic or SVs may have been missed. In this context, the increasing implementation of genome sequencing as a first-tier test in certain countries may further accelerate and streamline the diagnostic process [55]. Overall, this patient-centred, stepwise approach proved effective and can be adapted to routine clinical practice to optimise IRD diagnostics.

In conclusion, our case-by-case genomic approach significantly improved the diagnostic yield for IRDs. These findings support the routine integration of advanced sequencing methodologies, variant reclassification and functional validation in IRD diagnostics to optimise patient outcomes and expand the role of precision medicine in ophthalmic genetics. Future efforts should prioritise refining diagnostic workflows, identifying novel candidate genes, improving variant classification systems and incorporating emerging technologies such as long-read sequencing to further enhance diagnostic accuracy and patient care [47, 56].

## Summary

### What was known before:

- Whole-exome sequencing (WES) is the primary diagnostic tool for inherited retinal dystrophies (IRDs), yet approximately 40% of cases remain unresolved.
- Whole-genome sequencing (WGS) and functional assays have demonstrated potential to improve the diagnostic yield.
- Variants of uncertain significance (VUS) complicate clinical interpretation and limit patient access to gene therapies.

### What this study adds:

- A personalised genomic approach integrating WES reanalysis, WGS and customised gene panels improves IRD diagnosis.
- Deep intronic, non-coding and structural variants were identified, broadening the spectrum of IRD-related variants.
- Functional assays and systematic variant reclassification resolved previously undiagnosed cases.

## Supplementary information

Supplementary Material

Table S3

## Data Availability

The datasets generated during and/or analysed during the current study are available from the corresponding author on reasonable request.
