# Supplementary material for: Personalised genomic strategies improve diagnostic yield in inherited retinal dystrophies: a stepwise, patient-centred approach
Source: Eye (Lond). 2025 Sep 9;39(16):2899–911. doi: 10.1038/s41433-025-03981-1 (PMC12583820; doi:10.1038/s41433-025-03981-1)
Supplement: Supplementary file 1 — Supplementary Material [file 41433_2025_3981_MOESM1_ESM.docx]

Supplementary Material

1. Supplementary Data

List of 302 IRD genes analysed and corresponding transcripts used

*ABCA4* (NM_000350.3), *ABCC6* (NM_001171.6), *ABHD12* (NM_001042472.3), *ACBD5* (NM_145698.5), *ACO2* (NM_001098.3), *ADAM9* (NM_003816.3), *ADAMTS18* (NM_199355.4), *ADGRV1* (NM_032119.4), *AFG3L2* (NM_006796.3), *AGBL5* (NM_021831.6), *AHI1* (NM_001134831.2), *AIPL1* (NM_014336.5), *AIRE* (NM_000383.4), *ALDH3A2* (NM_000382.3), *ALMS1* (NM_001378454.1), *ALPK1* (NM_025144.4), *AMACR* (NM_014324.6), *ARHGEF18* (NM_001367823.1), *ARL13B* (NM_001174150.2), *ARL2BP* (NM_012106.4), *ARL3* (NM_004311.4), *ARL6* (NM_001278293.3), *ARSG* (NM_001267727.2), *ATF6* (NM_007348.4), *ATOH7* (NM_145178.4), *BBIP1* (NM_001195305.3), *BBS1* (NM_024649.5), *BBS10* (NM_024685.4), *BBS12* (NM_152618.3), *BBS2* (NM_031885.5), *BBS4* (NM_033028.5), *BBS5* (NM_152384.3), *BBS7* (NM_176824.3), *BBS9* (NM_198428.3), *BCOR* (NM_001123385.2), *BEST1* (NM_004183.4), *C1QTNF5* (NM_001278431.2), *C21orf2* (NM_004928.3), *C2orf71* (NM_001029883.3), *C8orf37* (NM_177965.4), *CABP4* (NM_145200.5), *CACNA1F* (NM_001256789.3), *CACNA2D4* (NM_172364.5), *CAPN5* (NM_004055.5), *CC2D2A* (NM_001378615.1), *CDH23* (NM_022124.6), *CDH3* (NM_001793.6), *CDHR1* (NM_033100.4), *CEP164* (NM_014956.5), *CEP250* (NM_007186.6), *CEP290* (NM_025114.4), *CEP41* (NM_018718.3), *CEP78* (NM_001330691.3), *CERKL* (NM_201548.5), *CFAP20* (NM_013242.3), *CFH* (NM_000186.4), *CHM* (NM_000390.4), *CIB2* (NM_006383.4), *CLCC1* (NM_001377458.1), *CLN3* (NM_001042432.2), *CLN5* (NM_006493.4), *CLN6* (NM_017882.3), *CLN8* (NM_018941.4), *CLRN1* (NM_174878.3), *CNGA1* (NM_001379270.1), *CNGA3* (NM_001298.3), *CNGB1* (NM_001297.5), *CNGB3* (NM_019098.5), *CNNM4* (NM_020184.4), *COL11A1* (NM_001854.4), *COL18A1* (NM_001379500.1), *COL2A1* (NM_001844.5), *COL4A1* (NM_001845.6), *COL9A1* (NM_001851.6), *COL9A2* (NM_001852.4), *COL9A3* (NM_001853.4), *COQ2* (NM_001358921.2), *COQ8B* (NM_024876.4), *CRB1* (NM_201253.3), *CRX* (NM_000554.6), *CSPP1* (NM_001382391.1), *CTC1* (NM_025099.6), *CTNNA1* (NM_001903.5), *CTNNB1* (NM_001904.4), *CTNND1* (NM_001085458.2), *CTSD* (NM_001909.5), *CWC27* (NM_005869.4), *CYP4V2* (NM_207352.4), *DHDDS* (NM_205861.3), *DHX38* (NM_014003.4), *DRAM2* (NM_001349884.2), *DYNC2H1* (NM_001377.3, NM_001080463.2), *EFEMP1* (NM_001039348.3), *ELOVL4* (NM_022726.4), *ERCC6* (NM_001277058.2, NM_000124.4), *ERCC8* (NM_000082.4), *EXOSC2* (NM_014285.7), *EYS* (NM_001142800.2), *FAM161A* (NM_001201543.2), *FAM57B* (NM_031478.6), *FBLN5* (NM_006329.4), *FLVCR1* (NM_014053.4), *FRMD7* (NM_194277.3), *FSCN2* (NM_012418.4), *FZD4* (NM_012193.4), *GDF6* (NM_001001557.4), *GNAT1* (NM_144499.3), *GNAT2* (NM_001377295.2), *GNB3* (NM_002075.4), *GNPTG* (NM_032520.5), *GPR143* (NM_000273.3 ), *GPR179* (NM_001004334.4), *GRK1* (NM_002929.3), *GRM6* (NM_000843.4), *GRN* (NM_002087.4), *GUCA1A* (NM_001384910.1), *GUCA1B* (NM_002098.6), *GUCY2D* (NM_000180.4), *HARS* (NM_002109.6), *HCCS* (NM_005333.5), *HGSNAT* (NM_152419.3), *HK1* (NM_000188.3, NM_001358263.1), *HKDC1* (NM_025130.4), *HMCN1* (NM_031935.3), *HMX1* (NM_018942.3), *IDH3A* (NM_005530.3), *IDH3B* (NM_006899.5), *IFT140* (NM_014714.4), *IFT172* (NM_015662.3), *IFT27* (NM_001177701.3), *IFT43* (NM_001102564.3), *IFT74* (NM_025103.4), *IKBKG* (NM_001099857.5), *IMPDH1* (NM_000883.4), *IMPG1* (NM_001563.4), *IMPG2* (NM_016247.4 ), *INPP5E* (NM_019892.6 ), *IQCB1* (NM_001023570.4), *KCNJ13* (NM_002242.4), *KCNV2* (NM_133497.4), *KIAA1549* (NM_001164665.2), *KIF11* (NM_004523.4), *KIF3B (NM_004798.4), KIZ* (NM_018474.6), *KLHL7* (NM_001031710.3), *LAMA1* (NM_005559.4), *LAMP2* (NM_002294.3), *LCA5* (NM_001122769.3), *LRAT* (NM_004744.5), *LRIT3* (NM_198506.5), *LRP2* (NM_004525.3), *LRP5* (NM_002335.4), *LZTFL1* (NM_020347.4), *MAK* (NM_001242957.3), *MCOLN1* (NM_020533.3), *MED12* (NM_005120.3), *MERTK* (NM_006343.3), *MFRP* (NM_031433.4), *MFSD8* (NM_001371596.2), *MKKS* (NM_170784.3), *MKS1* (NM_017777.4), *MMACHC* (NM_015506.3), *MPDZ* (NM_001378778.1), *MSTO1* (NM_018116.4), *MTTP* (NM_001386140.1), *MVK* (NM_000431.4), *MYO7A* (NM_000260.4), *NBAS* (NM_015909.4), *NDP* (NM_000266.4), *NEUROD1* (NM_002500.5), *NMNAT1* (NM_022787.4), *NPHP1* (NM_001128178.3), *NPHP3* (NM_153240.5), *NPHP4* (NM_015102.5), *NR2E3* (NM_014249.4), *NR2F1* (NM_005654.6), *NRL* (NM_001354768.3), *NYX* (NM_001378477.1, NM_001378477.3), *OAT* (NM_000274.4), *OFD1* (NM_003611.3), *OPA1* (NM_130837.3), *OPA3* (NM_025136.4), *OPN1LW* (NM_020061.6), *OPN1MW* (NM_000513.2), *OTX2* (NM_021728.4), *P3H2* (NM_018192.4), *PANK2* (NM_001386393.1), *PAX2* (NM_000278.5), *PCDH15* (NM_001384140.1, NM_033056.4), *PCYT1A* (NM_001312673.2), *PDE6A* (NM_000440.3), *PDE6B* (NM_000283.4), *PDE6C (NM_006204.4), PDE6G* (NM_002602.4), *PDE6H* (NM_006205.3), *PDSS1* (NM_014317.5), *PDZD7* (NM_001195263.2), *PEX1* (NM_000466.3), *PEX2* (NM_000318.3), *PEX6* (NM_000287.4), *PEX7* (NM_000288.4), *PHYH* (NM_006214.4), *PITPNM3* (NM_031220.4), *PLA2G5* (NM_000929.3), *PLK4* (NM_014264.5), *PNPLA6* (NM_001166114.2), *POC1B* (NM_172240.3), *POMGNT1* (NM_017739.4), *POMGNT2* (NM_032806.6), *POMT1* (NM_001077365.2), *PPT1* (NM_000310.4), *PRCD* (NM_001077620.3), *PRDM13* (NM_021620.4), *PROM1* (NM_006017.3), *PRPF3* (NM_004698.4), *PRPF31* (NM_015629.4), *PRPF4* (NM_001244926.2), *PRPF6* (NM_012469.4), *PRPF8* (NM_006445.4), *PRPH2* (NM_000322.5), *PRPS1* (NM_002764.4), *PYGM* (NM_005609.4), *RAB28* (NM_001017979.3, NM_004249.4), *RAX2* (NM_001319074.4), *RBP3* (NM_002900.3), *RBP4* (NM_006744.4), *RCBTB1* (NM_018191.4), *RD3* (NM_001164688.2), *RDH12* (NM_152443.3), *RDH5* (NM_002905.5), *REEP6* (NM_138393.4, NM_001329556.3), *RGR* (NM_001012720.2), *RGS9* (NM_003835.4), *RHO* (NM_000539.3), *RIMS1* (NM_014989.7), *RIMS2* (NM_001348484.3), *RLBP1* (NM_000326.5), *ROM1* (NM_000327.4), *RP1* (NM_006269.2), *RP1L1* (NM_178857.6), *RP2* (NM_006915.3), *RP9* (NM_203288.2), *RPE65* (NM_000329.3), *RPGR* (NM_001034853.2), *RPGRIP1* (NM_020366.4), *RPGRIP1L* (NM_015272.5), *RS1* (NM_000330.4), *RTN4IP1 (NM_032730.5), SAG* (NM_000541.5), *SAMD7* (NM_001304366.2), *SCAPER* (NM_020843.4), *SDCCAG8* (NM_006642.5), *SEMA4A* (NM_022367.4), *SGSH* (NM_000199.5), *SLC24A1* (NM_004727.3), *SLC38A8* (NM_001080442.3), *SLC6A6* (NM_003043.6), *SLC7A14* (NM_020949.3), *SNRNP200* (NM_014014.5), *SPATA7* (NM_018418.5), *SRD5A3* (NM_024592.5), *SSBP1* (NM_003143.3), *STN1* (NM_024928.5), *TEAD1* (NM_021961.6), *TIMM8A* (NM_004085.4), *TIMP3* (NM_000362.5), *TINF2* (NM_001099274.3), *TMEM216* (NM_001173990.3), *TMEM218* (NM_001258244.2), *TMEM231* (NM_001077418.3), *TMEM237* (NM_001044385.3), *TMEM67* (NM_153704.6), *TOPORS* (NM_005802.5), *TPP1* (NM_000391.4), *TRAF3IP1* (NM_015650.4), *TREX1* (NM_033629.6), *TRIM32* (NM_012210.4), *TRNT1* (NM_182916.3), *TRPM1* (NM_001252024.2), *TSPAN12* (NM_012338.4), *TTC8* (NM_144596.4), *TTLL5* (NM_015072.5), *TTPA* (NM_000370.3), *TUB* (NM_177972.3), *TUBB4B* (NM_006088.6), *TUBGCP4* (NM_014444.5), *TUBGCP6* (NM_020461.4), *TULP1* (NM_003322.6), *UNC119* (NM_005148.4), *USH1C* (NM_153676.4, NM_005709.4), *USH1G* (NM_173477.5), *USH2A* (NM_206933.4), *USP45* (NM_001346022.3), *VCAN* (NM_004385.5), *VPS13B* (NM_152564.5, NM_017890.5), *VWA8* (NM_015058.2), *WDPCP* (NM_015910.7), *WDR19* (NM_025132.4), *WHRN* (NM_015404.4), *ZFYVE26* (NM_015346.4), *ZNF408* (NM_024741.3), *ZNF423* (NM_001379286.1).

Candidate genes analysed and corresponding transcripts used

*CCZ1B* (NM_198097.5), *CROCC* (NM_014675.5), *FOXI2* (NM_207426.3), *FUT5* (NM_002034.2), *GARIN4* (NM_153606.4), *IRX5* (NM_005853.6), *ITIH2* (NM_002216.3), *NAALADL1* (NM_005468.3), *NUMB* (NM_001005743.2), *NUMBL* (NM_004756.5), *OR2M7* (NM_001004691.1), *OXR1* (NM_001198533.2), *PLD4* (NM_138790.5), *PODNL1* (NM_001370095.3), *POMZP3* (NM_012230.5), *PRDX6* (NM_004905.3), *PRTFDC1* (NM_020200.7), *SLC37A3* (NM_207113.3), *TLCD3B* (NM_031478.6), *UBAP1L* (NM_001163692.2), *WASF3* (NM_006646.6).

1. Supplementary Methods

***ABCA4* midigene-based splice assay**

The splicing effect of *ABCA4* c.859-442C>T was assessed using an *in vitro* splice assay, based on a previously established wild-type (WT) midigene (BA7) containing *ABCA4* exons 7 to 11 (1). The variant of interest was introduced into the WT construct via site-directed mutagenesis, followed by Gateway Cloning. Both constructs were transfected separately into Human Embryonic Kidney (HEK293T, ATCC# CRL-3216) cells, cultured in Dulbecco’s Modified Eagle Medium (DMEM) supplemented with 10% foetal bovine serum, 1% penicillin-streptomycin and 1% sodium pyruvate at 37°C and 5% CO_2_. Cells were seeded in a six-well plate and transfected at 70% confluency with 600 ng of plasmid DNA using FuGENE HD reagent (Promega, Madison, WI, USA), as per the manufacturer’s protocol. Transfection of the mutant construct was performed in duplicate. After 48 hours, total RNA was extracted using the Nucleospin RNA kit (Machery-Nagel, Düren, Germany) and cDNA was synthesised from 1000 ng of RNA using the iScript cDNA Synthesis kit (Bio-Rad, Hercules, CA, USA). Reverse transcription–polymerase chain reaction (RT-PCR) was performed to evaluate splicing. RT-PCR conditions for the *ABCA4* RNA were as follows: initial denaturation at 94°C for two minutes, followed by 30 cycles of 30 seconds at 94°C, 30 seconds at 58°C, and five minutes at 72°C, with a final extension step of two minutes at 72°C. *ACTB* (actin beta) was selected as housekeeping gene control and exon 5 of *RHO* served as a transfection control. RT-PCR conditions for these controls were: initial denaturation at 94°C for two minutes, followed by 35 cycles of 30 seconds at 94°C, 30 seconds at 58°C, and thirty seconds at 72°C and a final extension step of two minutes at 72°C.

Details on the primers used for mutagenesis, PCR, and Sanger sequencing are provided in Supplementary Table S2. Following PCR amplification, agarose gel analysis and Sanger sequencing were performed to assess the nature of splicing defects. Ratios between different RNA products were assessed by semi-quantification using Fiji software after agarose gel electrophoresis (Supplementary Table S5).

1. Supplementary Figures


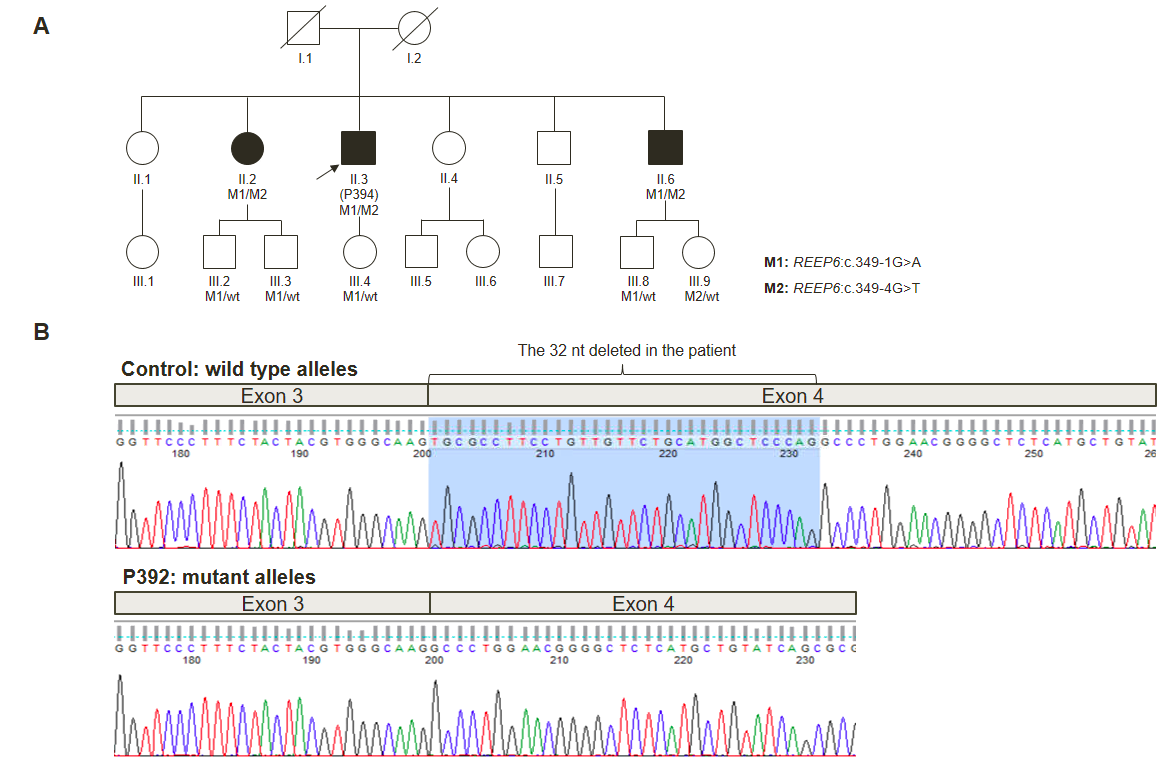


**Fig. S1. Effect of splicing variants c.349-1G>A and c.349-4G>T in *REEP6*. (A)** Pedigree of P394 illustrating variant co-segregation within the family. **(B)** Sanger sequencing electropherograms showing that both splice site variants lead to the deletion of 32 nucleotides from exon 4 of the *REEP6* gene.


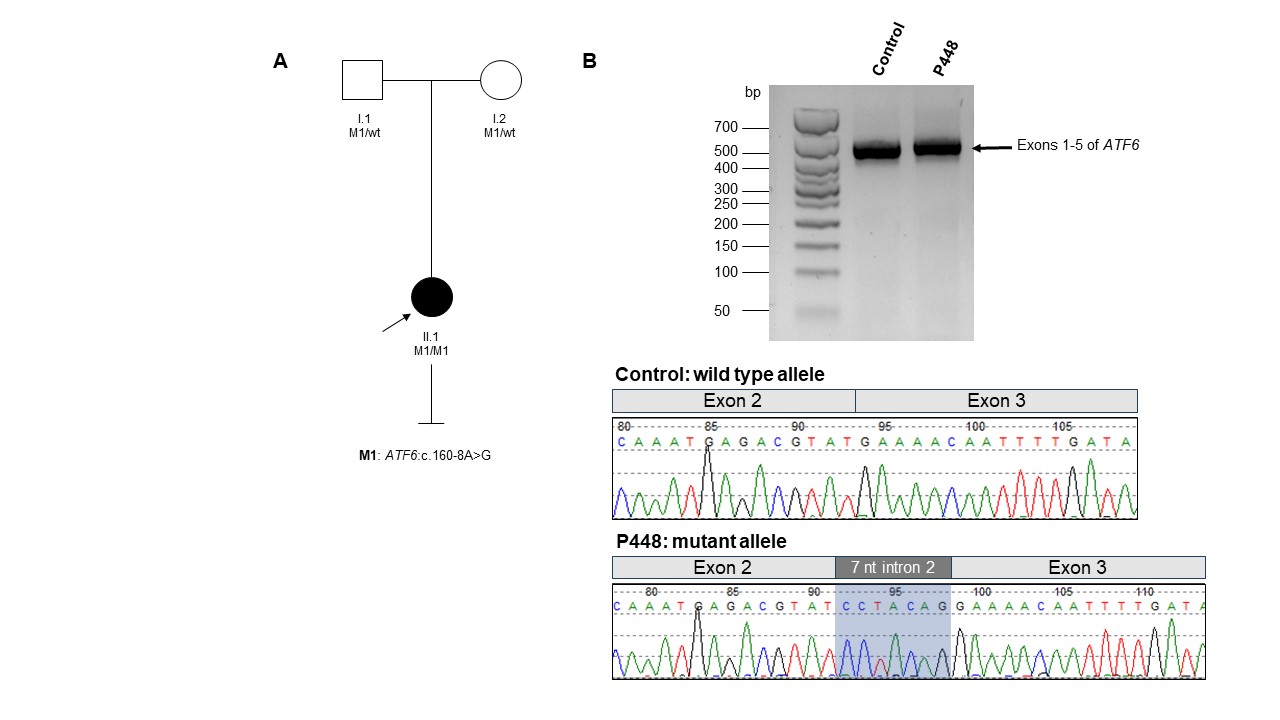


**Fig. S2. Splicing analysis of the c.160-8A>G variant in *ATF6* gene.** **(A)** Pedigree of P448 demonstrating that both parents are carriers of the c.160-8A>G variant. **(B)** Agarose gel analysis revealing a subtle increase in the band corresponding to the amplification of exons 1-5 of the *ATF6* gene in P448 compared to the control. Bellow, electropherograms illustrate the insertion of seven nucleotides from intron 2 into the coding sequence in P448 relative to the control.

1. Supplementary Tables

**Table S1.** Primers used for mRNA analysis of the variants identified in *ATF6* and *REEP6*.

| **Gene** | **Forward Primer (5'-3')** | **Reverse Primer (5'-3')** | **Product size (bp)** |
| --- | --- | --- | --- |
| *REEP6* | CACTCTGCTAAGCCTGTATC | CACTTGTCCTTCGGCTGC | 432 |
| *ATF6* | CACAGGCTGGATGAAGATTG | AGTCTTGTTCCTAGGACCAG | 424 |

**Table S2.** Oligonucleotides used in the splicing assay. **(A)** Primers for reverse transcription-PCR analysis and Sanger sequencing. **(B)** Oligonucleotides designed to introduce mutations into wild-type entry clones.

**A**

| **BA_ID** | **Primer sequences (5'-3')** | **Product size (bp)** |
| --- | --- | --- |
| BA7_forward | ACACTCCTAGACAGCCGTTC | 780 |
| BA7_reverse | CTCCAGGTATTGATTGACCAG |  |
| *RHO*_exon5_forward | ATCTGCTGCGGCAAGAAC | 140 |
| *RHO*_exon5_reverse | AGGTGTAGGGGATGGGAGAC |  |
| *ACTB*_mouse_exon3 | ACTGGGACGACATGGAGAAG | 383 |
| *ACTB*_mouse_exon4 | TCTCAGCTGTGGTGGTGAAG |  |

**B**

| **WT fragment** | **DNA variant_primer orientation** | **Mutagenesis primer sequences (5' - 3')** |
| --- | --- | --- |
| BA7 | c.859-442C>T_Fwd | CGATGTCTACTCACTGTAAGTTCTGCCCCCCAGG |
|  | c.859-442C>T_Rev | CCTGGGGGGCAGAACTTACAGTGAGTAGACATCG |

**Table S4.** In silico splicing predictions for candidate variants in *REEP6*, *ATF6* and *ABCA4*.

| **Gene** | **Variant** | **Splice AI score**  **Acceptor loss (position)** | **Splice AI score**  **Acceptor gain (position)** | **Splice AI score**  **Donor loss (position)** | **Splice AI score**  **Donor gain (position)** |
| --- | --- | --- | --- | --- | --- |
| *REEP6* | c.349-1G>A | 0.99 (1 bp) | 0.17 (33 bp) | - | 0.01 (212 bp) |
| *REEP6* | c.349-4G>T | 0.07 (4 bp) | 0.16 (36 bp) | - | - |
| *ATF6* | c.160-8A>G | 0.11 (8 bp) | 0.99 (1 bp) | - | - |
| *ABCA4* | c.859-442C>T | 0.01 (-304 bp) | 0.28 (243 bp) | - | 0.23 (6 bp) |

**Table S5.** Semi-quantification of RT-PCR fragments based on densitometry analysis of the gel electrophoresis results. Semi-quantification was performed on gel electrophoresis images using ImageJ software for two independent transfection experiments (biological replicates) and in technical replicates (1) and (2). PCR fragments are numbered as in Fig. 3. The average values were considered to determine the order of splice defects in the protein notation.

|  | **Mutant Fragment** | **Raw intensity**  **(1)** | **Raw intensity**  **(2)** | **Fragment size (nt)** | **Size correction factor** | **Corrected intensity**  **(1)** | **Corrected intensity**  **(2)** | **Percentage** | **Average (%)** |
| --- | --- | --- | --- | --- | --- | --- | --- | --- | --- |
| 1st biological replicate | 1 | 765,3 | 795,9 | 1.018 | 1,0 | 765,3 | 795,9 | 36,5% | **79,7% (MUT)** |
|  | 3 | 75,1 | 89,9 | 539 | 0,5 | 141,8 | 169,8 | 7,3% |  |
|  | 4 | 212,3 | 213,3 | 282 | 0,3 | 766,3 | 769,86 | 35,9% |  |
|  | 2 | 340,0 | 326,3 | 780 | 0,8 | 443,77 | 425,9 | 20,3% | **20,3%**  **(WT)** |
| 2nd biological replicate | 1 | 2543,9 | 2588,0 | 1.018 | 1,0 | 2543,9 | 2588,0 | 36,9% | **79%**  **(MUT)** |
|  | 3 | 273,7 | 280,1 | 282 | 0,3 | 516,9 | 529,1 | 7,5% |  |
|  | 4 | 669,4 | 663,9 | 539 | 0,5 | 2416,4 | 2396,9 | 34,6% |  |
|  | 2 | 1106,6 | 1130,9 | 780 | 0,8 | 1444,3 | 1475,9 | 21% | **21% (WT)** |

**References**

1. Sangermano R, Khan M, Cornelis SS, Richelle V, Albert S, Garanto A, et al. ABCA4 midigenes reveal the full splice spectrum of all reported noncanonical splice site variants in Stargardt disease. Genome Res. 2018 Jan;28(1):100–10.
